# Necessity for a Uniform Degree or Title

**Published:** 1895-02

**Authors:** 


Necessity for a Uniform Degree or Title.—Nothing is so mystifying to the public generally, and nothing so absurd to the intelligent public, as the many different applications which are affixed to the names of veterinarians. One is V. S., one D. V. S., another D. V. M., and still another V. M. D., while lately, that her graduates may still more deeply mystify the public, D. C. M. is brought forth by one of our colleges. Certainly if "simplicity is the mark of intelligence" our profession must be sadly wanting. In England it may be well enough and justifiable to add several letters to one's name, as the affix is uniform and well understood, and also carries with it the sole right to practise. In this country the only title that means anything is simply V. S., and why should we want anything more? It is plain, dignified, and brief. The quicker it is adopted as a uniform title by all of the colleges in the land the better for the profession. A man worthy of the confidence of the public should not try to mystify them, for if he does it will generally work, and justly so, to his disadvantage. It is an evidence not only of ignorance but of bad manners. We hope to see the time when every graduate practitioner will have printed upon his door-plate simply the letters V. S., or better the words "Veterinary Surgeon." The association of the veterinary faculties at present organized will do well, we believe, to adopt this simple title as one more becoming to the profession than D. V. S., D. V. M., V. M. D., or D. C. M.

